# Malignant Transformation Six Months after Removal of Intracranial Epidermoid Cyst: A Case Report

**DOI:** 10.1155/2011/525289

**Published:** 2011-09-25

**Authors:** Fayçal Lakhdar, El Mehdi Hakkou, Rachid Gana, Rachid My Maaqili, Fouad Bellakhdar

**Affiliations:** Department of Neurosurgery, Ibn Sina Hospital, Rabat, Morocco

## Abstract

Intracranial epidermoid cysts are uncommon benign tumors of developmental origin; malignant transformation of benign epidermoid cysts is rare, and their prognosis remains poor. We report a case of squamous cell carcinoma arising in the cerebellopontine angle. A 52-year-old man presented with left facial paralysis and cerebellar ataxia. He had undergone total removal of a benign epidermoid cyst six months previously. Postoperative magnetic resonance imaging of the brain revealed a heterogeneous and cystic lesion in the left cerebellopontine angle with hydrocephalus. The cyst wall was enhanced by gadolinium. He underwent ventricle-peritoneal shunt and removal again; the histopathological examination revealed a squamous cell carcinoma possibly arising from an underlying epidermoid cyst. This entity is being reported for its rarity. The presence of contrast enhancement at the site of an epidermoid cyst combined with an acute, progressive neurological deficit should alert the neurosurgeon to the possibility of a malignant transformation.

## 1. Introduction

Intracranial epidermoid cysts (ECs) are rare, histologically benign, slow-growing, congenital neoplasms of the central nervous system that may arise from retained ectodermal implants [11]. Malignant transformation of an EC to squamous cell carcinoma (SCC) is rare; only 10 cases have been reported. Here we describe an exceptional case of EC in the cerebellopontine angle (CPA) which underwent malignant transformation six months only after removal.

## 2. Case Report

**Figure 1:**
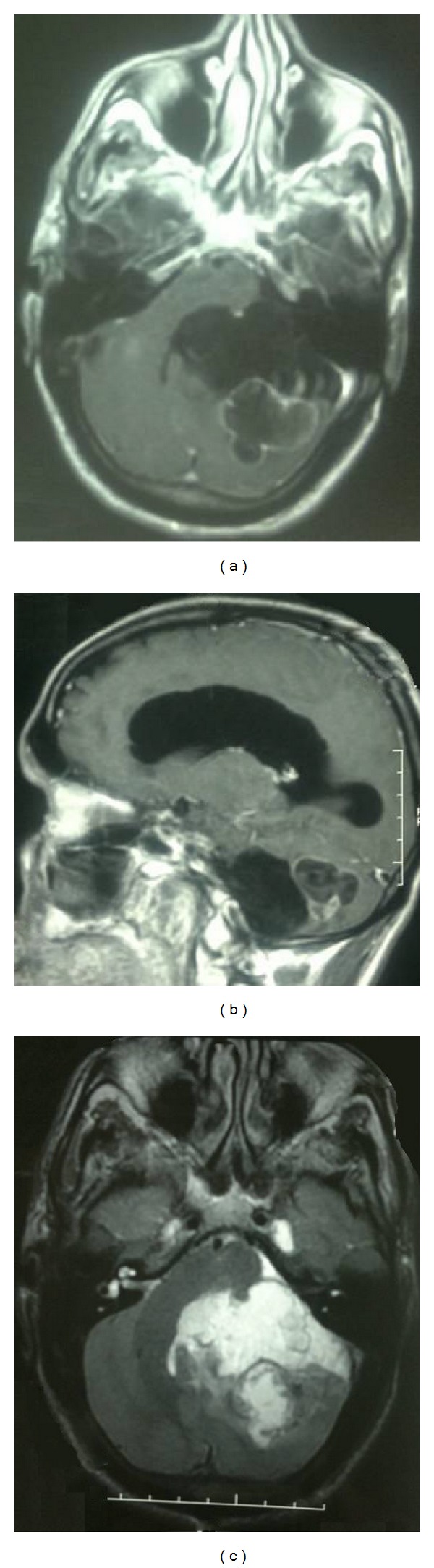
Axial (a) and sagittal (b) T1-weighted MRI with gadolinium and axial (c) T2-weighted MRI before surgery revealing a large cystic lesion of left CPA with enhancement of lower portion of the lesion and severe compression of forth ventricle and brainstem.

A 52-year-old man had first presented with dizziness, cerebellar ataxia, and facial paresis (grade 3 House-Brackmann) (HB3). Magnetic resonance imaging (MRI) revealed a large cystic lesion of CPA, hypointense on T1-weighted image, and hyperintense on T2-weighted image, extending to the left parapontine, with partial enhancement of the capsule after intravenous administration of gadolinium (Figure 1).

The tumor was removed via the suboccipital retrosigmoid approach in the sitting position, and surgery revealed a large cyst with a thin layer of white capsule containing yellowish-white, soft, and cheesy material. The cyst including the capsule and contents was removed completely. The patient recovered without incident, and the histological diagnosis was EC.

**Figure 2:**
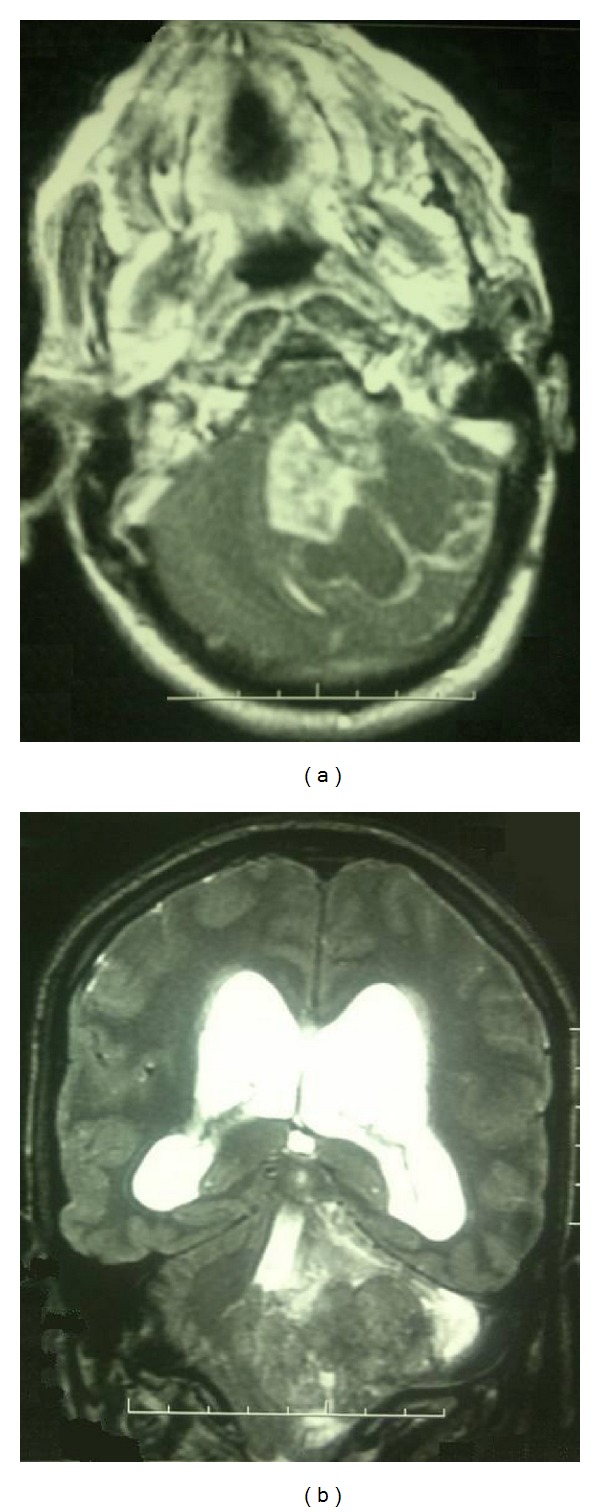
Axial (a) T1-weighted MRI with gadolinium and coronal; (b) T2-weighted MRI after the first surgery showing growth of the heterogeneous lesion with predominant enhancement and invading the surrounding structures.

**Figure 3:**
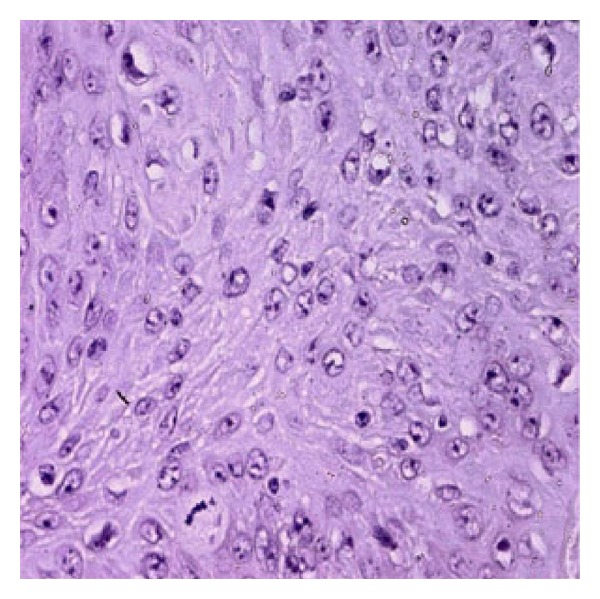
Malignant squamous cell carcinoma arising from the epidermal cyst, with mitotic activity and cellular atypia (hematoxylin and eosin, original magnification ×400).

Six months after the first surgery, he again presented severe facial paresis (HB5), gait disturbance, and intracranial pressure (ICP). MRI demonstrated a large heterogeneous mass isointense on T1-weighted image, hyperintense on T2-weighted image, strongly enhanced after gadolinium, extending to the upper medulla oblongata with hydrocephalus and unusual edema (Figure 2). Rapid deterioration of his neurological symptoms associated with the location of the enhanced lesion in MRI suggests malignant transformation of EC. He underwent ventricle-peritoneal shunt in emergency followed by second total resection of the recurrent tumor, and histological examination of the specimen showed a cystic lesion lined by bland squamous epithelium and filled with laminated keratin. There were several small scattered islands of severely atypical squamous epithelium. These areas of typical epidermal cyst were juxtaposed with zones that displayed marked nuclear irregularity with mitotic activity and an infiltrative growth pattern (Figure 3). Immunohistochemistry showed positivity of the tumor cells for P53 protein. Based on these findings, the diagnosis of squamous cell carcinoma (SCC) arising in an EC was made. 

Local radiation therapy (50 Gy) was administrated after surgery, with good followup, one month later his facial paralysis was well (HB2) without ICP.

## 3. Discussion

Epidermoid or “pearly” tumors were described by Cruveilhier and designated the “most beautiful tumors of the body” by Dandy. Although the formation of epidermoid lesions develop from aberrant ectodermal embryonic tissue in the neural groove at 4 or 5 weeks of fetal development, the mechanical introduction of such skin elements can also occur by skin puncture [12].

Malignant transformation of EC into carcinoma is quite rare and was first reported by Ernst in 1912 [13]. Most previous cases of these carcinomas were discovered within benign EC at the first surgery or autopsy [14]. Malignant transformation from remnant EC long after the initial surgery is extremely rare reported in only 10 cases (Table 1). The patients were aged from 36 to 74 years (mean 53 years) with female predominance. The interval from first operation to malignant transformation ranged from 2 to 33 years (mean 14 years). Our case is the shortest period of malignant transformation in the literature (six months only). However, SCCs were probably already present in most cases.

Rapid progression of symptoms and signs is the most important clinical indication of malignant transformation of EC. When transformation does occur, the clinical and radiological course is quite aggressive as compared with the indolent growth of EC [10]. The typical MRI features of EC include irregular shape, good demarcation, low intensity, and absence of edema. Most of EC are not enhanced by gadolinium, but minimal rim enhancement occurs in approximately 25% of cases [2, 7]. Calcification is rare in EC and is usually marginal, probably due to the peritumoral leak of the contents with secondary dystrophic calcification. In Our case, the initial EC had these MRI features, whereas the recurrent EC appeared as hyperintense. While the presence of a cystic area limited by segments of benign squamous cell epithelium is required for the diagnosis of malignant transformation in a preexisting EC, this diagnosis should also be retained when MRI shows contrast enhancement at the epidermoid site and malignant cells are detected in CSF [6, 7]. However, malignant transformation should be considered when follow-up MRI shows contrast enhancement at the surgical site, in any patient with a known benign cyst, who does not make the expected recovery, and/or whose condition deteriorates.

In the present case, the rapid deterioration of symptoms was concomitant with predominant enhancement of the tumor on MRI, thus suggesting extremely rare malignant transformation of an IEC. The mechanisms of malignant transformation of EC are controversial and may involve inflammation caused by reaction to foreign bodies or in situ carcinoma. Chronic inflammatory response to repeated cyst rupture and subtotal resection of the cyst wall may cause the malignant transformation [10, 14]. So, the malignant transformation of the EC in the current case cannot be explained by only inflammation, because long-standing inflammation was not observed.

Treatment options include surgery with adjuvant chemotherapy or radiotherapy. Radiation therapy for intracranial SCC has been effective over a short period and stereotactic radiation therapy following the surgery is also effective in some cases, with disease-free survival of more than 5 or 8 years or local tumor control for 29 months [8, 14]. Recently, Gamma Knife neurosurgery for adjuvant therapy was also reported as useful [8].

## 4. Conclusion

Rapid onset of symptoms, recurrence, leptomeningeal carcinomatosis, and tumor enhancement in MRI suggests malignant degeneration of the EC. Surgery resection and adjuvant radiation therapy are highly recommended.

## Figures and Tables

**Table 1:** Cases of malignant transformation of epidermoid cyst.

Case no.	Author (year)	Age/sex	Tumor location	Treatment	Interval (months)	Postoperative course
1	Fox and South (1965) [1]	43/M	Temporal	Surgery	84	Died 1 month
2	Goldman and Gandy (1987) [2]	59/F	Intraventricular	Surgery, radiation therapy	396	Alive 36 months
3	Abramson et al. (1989) [3]	36/M	CP angle	Surgery	24	Not described
4	Tognetti et al. (1991) [4]	67/F	Frontotemporal	Surgery	372	Died 1 month
5	Murase et al. (1999) [5]	50/F	CP angle	Surgery, chemotherapy, radiation therapy	120	Alive 60 months
6	Asahi et al. (2001) [6]	55/F	CP angle	Surgery	156	Died 3 months
7	Nawashiro et al. (2001) [7]	46/F	Temporal	Surgery	?	Not described
8	Tamura et al. (2006) [8]	56/F	CP angle	Surgery, Gamma Knife	96	Alive 13 months
9	Ge et al. (2009) [9]	50/M	Temporal	Surgery	72	Not described
10	Nakao et al. (2010) [10]	74/F	CP angle	Surgery, radiation therapy	240	Alive 17 months
11	Present case Lakhdar et al. (2011)	52/M	CP angle	Surgery, radiation therapy	6	Good (3 months followup)

## Abbreviations

EC: epidermoid cyst
MRI: Magnetic resonance imaging
SCC: Squamous cell carcinoma
CPA: Cerebellopontine angle
HB: House -Brackmann
ICP: intracranial pressure.
